# The Promoting Healthy Sleep E-Learning Professional Development Program for Health Care Providers: Mixed Methods Usability Study

**DOI:** 10.2196/91150

**Published:** 2026-08-11

**Authors:** Alzena Ilie, Elizabeth Keys, Alissa Pencer, Shelly Weiss, Penny Corkum

**Affiliations:** 1 Department of Psychology and Neuroscience Faculty of Science Dalhousie University Halifax, NS Canada; 2 School of Nursing University of British Columbia Okanagan, BC Canada; 3 Department of Psychiatry Faculty of Medicine Dalhousie University Halifax, NS Canada; 4 Department of Paediatrics Faculty of Medicine University of Toronto Toronto, ON Canada

**Keywords:** e-learning program, health care providers, insomnia, pediatrics, professional development, school-aged, sleep, usability study

## Abstract

**Background:**

Approximately one-third of Canadian children have insomnia or symptoms of insomnia, defined as chronic and frequent difficulties falling and/or staying asleep. Most health care providers (HCPs) in Canada do not receive comprehensive formal training in the screening, assessment, or management of pediatric insomnia.

**Objective:**

This study examined the perceived usability of an e-learning professional development program for HCPs, titled Promoting Healthy Sleep (PHS). The goal of this program is to provide accessible online sleep education to HCPs to help inform their screening, assessment, and treatment of pediatric insomnia in children aged 5 to 12 years.

**Methods:**

For this usability study, 22 HCPs working in Canada were recruited from 4 groups (ie, physicians, psychologists, nurses, and allied health professionals), and they assessed the usability of the program using the UX Honeycomb framework from the end users’ perspective. Questionnaires that evaluated program usability, satisfaction, and acceptability, as well as participants’ sleep knowledge and sleep attitudes and beliefs, were administered.

**Results:**

HCPs’ sleep knowledge *(P*<.001), as well as their attitudes and beliefs (*P*=.02), significantly improved from before to after program completion. HCPs provided high satisfaction ratings and found the program to be highly usable (ratings of 80% or higher on all program usability and satisfaction questionnaires). They also provided minor suggestions about content that should be revised in the program, such as the need for additional resources and program content suggestions (eg, modifications to increase user-friendliness, program content suggestions, and adding additional resources for HCPs).

**Conclusions:**

Study participants found the PHS program usable and were satisfied with the program content. Accessible HCP training will lead to better identification and treatment of insomnia in school-aged children, as HCPs will be better equipped and more knowledgeable about how to treat insomnia with the proper steps.

## Introduction

Pediatric insomnia is one of the most common disorders reported in children [1]. In the general Canadian population, it is estimated that approximately 30% of children have symptoms of insomnia, including trouble falling or staying asleep, with 5% to 20% of children meeting diagnostic criteria for insomnia disorder, including frequent (3 or more times a week) and chronic (more than 3 months) difficulties with insomnia symptoms that impair their daytime functioning [2-4]. Insomnia disorder/insomnia symptoms are associated with increased daytime sleepiness, fatigue, reduced alertness, poorer neurobehavioral functioning, and negative effects on cognitive functioning and emotional regulation [5-7]. Insufficient sleep is also associated with increased rates of mental health disorders, deficits in learning and academic performance, and negative effects on physical health, including increased rates of metabolic disorders and obesity.

Insomnia disorder/insomnia symptoms (herein referred to as insomnia) in children can be managed within a stepped care model [8]. The first step is sleep education, which involves providing information to parents (and children, if developmentally appropriate) about the purpose of sleep, the biological processes associated with sleep, what constitutes adequate amounts of sleep, and the negative consequences associated with insufficient sleep or sleep deprivation [9,10]. The second step involves focusing on healthy sleep practices and promoting healthy behaviors that facilitate sleep (eg, consistent bed and wake times and bedtime routines) while avoiding behaviors that impede sleep (eg, using technology before bedtime) [9,11,12]. The third step consists of strategies that are based on learning and behavioral principles, such as sleep scheduling, paired associative learning, and graduated/total extinction [9,11-13]. The final step involves prescribing sleep medication and/or supplements, such as melatonin, or referring the child to a more advanced practitioner (eg, a sleep specialist) [9,11,13]. Medication, whether prescribed or over-the-counter, should typically be recommended only after all other steps have been tried and found to have limited effectiveness. Prescription and monitoring of medication should be conducted by a prescribing HCP with expertise in sleep medicine [14,15].

While these steps involved in treating pediatric insomnia are well established, few health care providers (HCPs) have the education and resources required to provide the sequence of steps described above. It has been found that most prescribing HCPs start addressing sleep concerns with medication rather than with psychoeducational and behavioral strategies, which are considered the first-line treatment for pediatric insomnia and sleep disturbances [16]. For example, Bock et al [17] found that two-thirds of family physicians and over three-quarters of pediatricians in Ontario recommended sedating medication over a 6-month period to treat insomnia among children. In another sample that consisted primarily of physicians, almost 50% of participants who worked with children who had insomnia recommended melatonin over behavioral interventions as the first-line treatment [18]. This is not surprising, as most HCPs who work with children do not receive comprehensive formal training in screening, assessment, or the management of insomnia in Canada [17,18] or elsewhere [19,20]. In fact, a survey provided to 97 Canadian pediatric HCPs (with the majority of participants being physicians) revealed that only 3% of HCPs received formal sleep training in this area [18]. Moreover, the Canadian Pediatric Society does not recommend melatonin as a first-line treatment for typically developing children with insomnia symptoms [16]. These studies highlight the gap between evidence-based recommended care and actual practice in the treatment of pediatric insomnia (ie, the evidence-to-practice gap) [3].

In the landscape of health care, the lack of pediatric sleep training programs for HCPs in Canada and elsewhere underscores the critical need for these professional development programs [20]. To address the evidence-to-practice gap in HCPs’ insomnia training, an e-learning professional development program, called the Promoting Healthy Sleep (PHS) program, was created. In user-centered design, the program is tailored to end users’ needs and ensures that users are using the program as intended with minimal effort to use and navigate the program [21,22]. The initial phase involves evaluating its usability through an iterative process [21,22]. If there are favorable usability ratings, the next step is to test its effectiveness (eg, conducting randomized controlled trials). Once proven efficacious, the final stage involves evaluating the implementation of the program within routine practice [23]. This iterative process ensures that the program is tailored to end users’ specific needs and also supports a smooth integration within health care practices.

**Figure 1:**
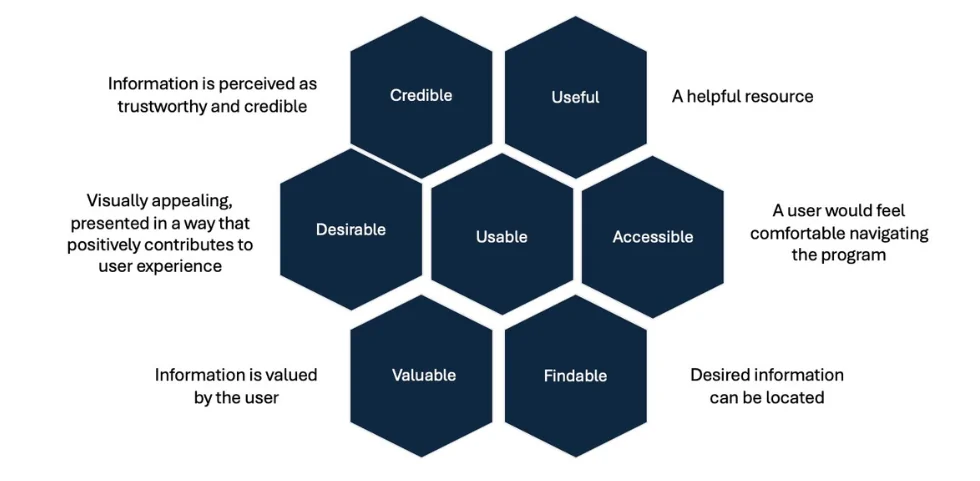
UX Honeycomb framework. It is based on Morville and Sullenger’s [24] 2010 UX Honeycomb framework. These 7 elements affect user experience.

To gather input from end users, this study engaged HCPs, including physicians, pediatricians, psychologists, nurses, and allied health professionals, to evaluate the usability of the PHS program. The HCPs reviewed the PHS program and provided quantitative and qualitative feedback on the program’s usability. We used the UX Honeycomb framework developed by Morville and Sullenger [24] to guide the user-centered assessment and to understand the factors that impact the desire and motivation to use the PHS program. This theoretical framework includes 7 distinct dimensions of usability: usable, useful, desirable, findable, valuable, accessible, and credible (Figure 1 [24]).

The research objectives of the present study are as follows:

- To assess HCPs’ ratings of overall satisfaction with the PHS program.
- To assess HCPs’ ratings of overall program usability for each of the 7 user dimensions (including useful, usable, findable, credible, valuable, accessible, and desirable).
- To assess HCPs’ usability ratings (including all 7 honeycomb framework dimensions) for each session.
- To assess participants’ ratings of the acceptability of content across each session and for the program overall.
- To gather qualitative constructive feedback about the program from HCP participants.
- To assess the preliminary effectiveness of the PHS program in terms of improving HCP knowledge about the nature, assessment, and treatment of pediatric insomnia, and changing attitudes and beliefs about sleep in children.

We expect that the majority of HCPs (>80%) will have high overall satisfaction ratings with the program, as well as high ratings for usability and program acceptability. We also anticipate that there will be a significant difference in knowledge scale scores and sleep attitudes and beliefs scores from preintervention to postintervention completion. Additionally, we expect to receive program content suggestions and changes reported by participants in order for the program to better meet their needs.

## Methods

### Study Design

This usability pilot study used a mixed methods pre-post convergent design to evaluate the usability and preliminary effectiveness of the PHS program among health care providers from diverse disciplines. In this design, quantitative (eg, questionnaire ratings and pre-post outcomes) and qualitative (eg, open-ended feedback) data were collected concurrently and integrated during interpretation to provide a more comprehensive understanding of the user experience.

### PHS Program

**Figure 2:**
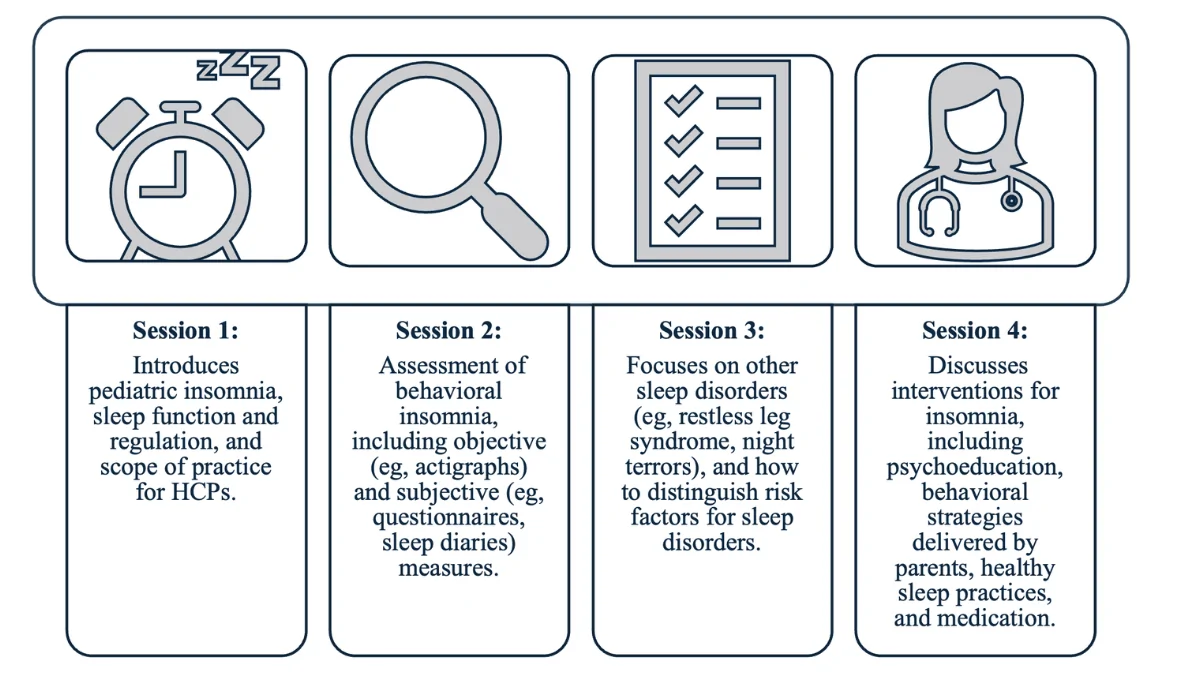
Promoting Healthy Sleep program session content. HCP: health care provider.

The PHS program was developed by an interdisciplinary team of investigators, including PC (psychology), SW (medicine), and EK (nursing). The PHS professional development program includes 4 sessions, each approximately 30 minutes in duration, which HCPs can complete in approximately 2 hours. The program focuses on assessing pediatric insomnia in children aged 5 to 12 years, information about other pediatric sleep disorders, and how to evaluate and manage insomnia in children (Figure 2 provides a description of each session). To ensure ease of access, scalability, and cost-effectiveness, the PHS program was created as an e-learning program. E-learning programs are delivered online via the internet and have the potential to reduce access barriers. Multiple studies have examined the delivery of e-learning programs, and e-learning has been shown to be effective in delivering professional development programs to HCPs [25-28].

### Participants

For this study, our goal was to recruit 20 HCPs practicing in Canada whose general practice includes working with school-aged children, some of whom have insomnia. Eligible participants had to (1) be fluent in English (as the program is currently available only in English), (2) be a regulated HCP (ie, part of a regulatory body), and (3) have worked over the past 2 years with a minimum of 5 children aged 5 to 12 years for whom the primary concern expressed by the parent was that their child had symptoms of insomnia. Participants were ineligible for the study if they were considered experts in pediatric sleep, worked in a clinical practice that was mainly focused on pediatric sleep disorders, or were sleep coaches/consultants but not regulated HCPs. Our goal was to recruit 5 participants in each of the 4 targeted HCP groups: physicians (eg, family physicians, pediatricians, and psychiatrists), psychologists, nurses, and allied health professionals (eg, social workers, occupational therapists, and recreational therapists). In previous research, it has been suggested that about 85% of unique feedback and problems that are inherent in usability studies can be detected with only 5 participants [29], so the sample size of 5 participants for each of the 4 HCP groups should detect problems that need to be addressed in the PHS program.

### Procedures

A multipronged recruitment strategy was used, and recruitment was conducted from June to November 2023. The investigators recruited directly through their professional and disciplinary networks for this study. Advertising to recruit participants was done through hospital and professional body newsletters throughout Canada and was also advertised on LinkedIn, Instagram, Twitter, and Facebook.

Potential participants were provided with a link to a brief electronic screening questionnaire administered on REDCap [30] to ensure that they met the criteria for the study and to confirm the professional body with which they were registered to verify that they were licensed HCPs. Once their status as a licensed HCP was confirmed by a researcher (AI), eligible participants were automatically directed to the electronic Information and Consent Form. Once consent had been provided, participants were directed to complete the demographic questionnaire, sleep attitudes and beliefs questionnaire, and sleep knowledge questionnaire. HCPs who completed these steps were given access to the e-learning program for 1 month.

Session content was delivered via the internet in a lecture-style video format (ie, a speaker presenting program content with slides) and was displayed sequentially (following mandatory completion of the previous session), with each video session lasting approximately 30 minutes (Figure 2 provides the PHS session content). This usability study consisted of asking HCPs to provide feedback on their perceptions of the content and delivery of the program and their personal likelihood of using these materials in their respective work settings to support their work in assessing and treating pediatric insomnia. Once all 4 sessions were completed and all study questionnaires were completed, participants received a certificate of completion that they were able to download for their professional development portfolio.

### Measures

#### Screening Questionnaire

This is an author-made, 5-item questionnaire that was used to verify that HCPs were eligible to participate in this study. The questionnaire had items pertaining to the eligibility criteria for the study (eg, specific criteria are provided in the Participants section).

#### Demographic Questionnaire

This is an author-made, 14-item questionnaire that was used to collect demographic information about the study sample, including HCPs’ location within Canada, gender, and education. Participants also answered questions related to pediatric insomnia, including a rating of perceived knowledge about pediatric insomnia (eg, “What is your perception of your ability to evaluate and treat sleep problems in children?”), the proportion of children with insomnia in their practice over the past 2 years, details about previous education/courses on pediatric sleep, and motivation for participating in the study.

#### Satisfaction Questionnaire

The Satisfaction Questionnaire (SQ) is an author-made questionnaire developed based on the categories of the Client Satisfaction Questionnaire-8 (CSQ-8; eg, quality of service, outcome of service, and general satisfaction) [31]. It consisted of 8 items on a 4-point Likert scale (ie, 1=no, definitely not; 4=yes, definitely). The SQ gathered information on the perceived quality, usefulness, and satisfaction with the program. A textbox was included after each rating to collect additional qualitative feedback on the program.

#### Acceptability and Usability Questionnaires

##### Program Acceptability and Usability Questionnaire

The 11-item Program Acceptability and Usability Questionnaire (P-AUQ) asked HCPs to give feedback about the perceived usability of the program overall. This author-made questionnaire was adapted from a questionnaire used in a previous study [32] and was designed based on Morville and Sullenger’s [24] UX Honeycomb framework and assessed the 7 aforementioned dimensions (Figure 1). Participants were asked to provide ratings between 1 and 5 (eg, 1=strongly disagree; 5=strongly agree) for each of the 7 user dimensions in relation to the PHS program and were asked to provide commentary to explain their ratings within each category in the textbox below each rating. In addition to the above ratings, the questionnaire included questions regarding technical issues experienced, whether any information/materials should be added, removed, or changed within the program, whether the program was ready to use, and participants’ overall experience with the e-learning program. The P-AUQ was completed after all 4 sessions were reviewed.

##### Session Acceptability and Usability Questionnaire

The author-made 6-item Session Acceptability and Usability Questionnaire (S-AUQ) asked HCPs to give feedback about the perceived usability of each of the 4 sessions according to the honeycomb user constructs (Multimedia Appendix 1 provides a copy of this questionnaire) [24]. The questionnaires were completed by participants sequentially as they reviewed each of the 4 sessions of the program. The questionnaire included the following feedback on the sessions: whether any information should be added to, removed from, or changed in the sessions and whether the sessions were ready for use. Participants were asked to provide ratings (eg, 1=strongly disagree; 5=strongly agree) for each of the 7 honeycomb user dimensions of the PHS sessions and were asked to provide commentary to explain their ratings within each category in the textbox below each rating.

#### Sleep Knowledge Questionnaire

The author-made Sleep Knowledge Questionnaire (SKQ) is a 21-item measure that was intended to characterize HCPs’ knowledge of pediatric insomnia (Multimedia Appendix 1 provides a copy of this questionnaire). At the time of this study, there were no sleep knowledge questionnaires in the literature to assess knowledge for a multidisciplinary group of HCPs, so one was created for this study. The SKQ consisted of 18 questions with Likert-scale response options and 3 true-or-false questions. The SKQ was totaled for an overall score. The sessions in the PHS program were used to create general sleep knowledge questions so that an overall assessment of sleep knowledge could be obtained. The SKQ was completed before the start of the program and after the program was completed.

#### Sleep Attitudes and Beliefs Questionnaire

The author-made Sleep Attitudes and Beliefs Questionnaire (SABQ) is a 17-item measure that was intended to characterize HCPs’ attitudes and beliefs about sleep. This questionnaire, originally designed to be used with parents, was modified to be completed by HCPs in order to measure their attitudes and beliefs about sleep. The SABQ was completed before the start of the program and after the program was completed. The Cronbach α values for each subscale were as follows: sleep modifiability (α=0.54), responsiveness to treatment (α=0.56), sleep impact (α=0.78), and nature of sleep problems (α=0.86). These values suggest moderate to good internal consistency for the latter 3 subscales and fair internal consistency for the sleep modifiability subscale.

### Data Analysis

For quantitative data, SPSS (version 28; IBM) was used to conduct all analyses. Descriptive statistics (means and SDs for continuous variables; percentages for categorical variables) were calculated for the HCP demographic questionnaire, the S-AUQ and P-AUQ, and the SQ. Paired 2-tailed *t* tests were conducted for the SKQ and SABQ (both administered preintervention and postintervention to HCPs), and effect sizes for both questionnaires were calculated using Cohen *d*.

Qualitative data from the P-AUQ, S-AUQ, and SQ were managed using Microsoft Excel and analyzed using inductive content analysis [33]. Content analysis involves three stages: (1) preparing the data (eg, deciding what units of data to code), (2) organizing the data (eg, open coding and creating categories), and (3) reporting themes (eg, discussion of the analysis process and results). Qualitative data were inductively coded into themes by the primary author (AI) and were checked for rigor and trustworthiness by a senior member of the research team (PC). For the HCP qualitative data, a theme was required to be identified by 3 or more individual participants, as established a priori for threshold inclusion (3/22, 14%) [34,35]; an a priori theme threshold has been used in similar qualitative studies evaluating programs [34-36].

### Ethical Considerations

The study involved human participants, and it was reviewed and approved by the Izaak Walton Killam Hospital Ethics Review Board (#1027643). The participants provided written informed consent to participate in this study, and all study data were anonymized. Participants received a CAD $50 (CAD $1=US $0.76 as of June 20, 2023) gift card honorarium for completing the study. The study adhered to the Declaration of Helsinki. A clinical trial number was not applicable.

## Results

### Sample Characteristics

Out of the 28 HCPs who consented to participate, 22 HCPs completed the study, which was slightly above our target of 20 participants and represents a 79% retention rate. Six participants withdrew because they did not have the time commitment available to complete the study. All those who participated in the study (N=22; 100%) reviewed the entire (ie, all 4 sessions) PHS program, and there were no missing data. Table 1 provides the participant-reported demographic characteristics and demographic characteristics divided by HCP occupation group.

**Table 1:** Demographic details reported by HCP^a^ type.

Primary workplace setting			All participants^b^ (N=22), n (%)		Physicians (n=5), n (%)		Psychologists (n=6), n (%)		Nurses (n=5), n (%)		Allied health professionals (n=6), n (%)
Private practice			8 (36.4)		2 (40)		4 (66.7)		0 (0)		2 (33.3)
Community health/mental health center			5 (22.7)		1 (20)		2 (33.3)		0 (0)		2 (33.3)
Outpatient/community-based clinic			4 (18.2)		2 (40)		0 (0)		2 (40)		0 (0)
Hospital			3 (13.6)		0 (0)		0 (0)		3 (60)		0 (0)
School/not-for-profit education program			2 (9.1)		0 (0)		0 (0)		0 (0)		2 (33.3)
**Gender**											
	Women	21 (95.5)		5 (100)		6 (100)		5 (100)		5 (83.3)	
	Men	1 (4.5)		0 (0)		0 (0)		0 (0)		1 (16.7)	
**Highest education**											
	Doctorate (ie, PhD)	2 (9.1)		0 (0)		2 (33.3)		0 (0)		0 (0)	
	Medical degree (ie, MD)	5 (22.7)		5 (100)		0 (0)		0 (0)		0 (0)	
	Master’s degree	11 (50)		0 (0)		4 (66.7)		3 (60)		4 (66.7)	
	Bachelors/undergraduate degree	4 (18.2)		0 (0)		0 (0)		2 (40)		2 (33.3)	
**Experience working as an HCP (years)**											
	1-10	10 (45.5)		2 (40)		2 (33.3)		2 (40)		4 (66.7)	
	11-20	6 (27.3)		0 (0)		3 (50)		2 (40)		1 (16.7)	
	>20	6 (27.3)		3 (60)		1 (16.7)		1 (20)		1 (16.7)	
**Experience working with school-aged children as an HCP (years)**											
	1-10	10 (59.1)		2 (40)		2 (33.3)		2 (40)		4 (66.7)	
	11-20	6 (27.2)		0 (0)		3 (50)		2 (40)		1 (16.7)	
	>20	6 (27.2)		3 (60)		1 (16.7)		1 (20)		1 (16.7)	
**Time working with school-aged children (%)**											
	5-30	6 (27.3)		0 (0)		1 (16.7)		3 (60)		2 (33.3)	
	40-70	11 (50)		5 (100)		3 (50)		1 (20)		2 (33.3)	
	80-100	5 (22.7)		0 (0)		2 (33.3)		1 (20)		2 (33.3)	

^a^HCP: health care provider.

^b^Percentages in the “all participants” column were calculated using the total sample size (N=22).

When participants were asked about their perceptions of their ability to evaluate and treat sleep problems in children before completing the program, 82% (18/22) of participants stated that they felt they were able to evaluate/treat sleep problems but not as well as they would like, and the remaining 18% (4/22) stated that they felt they were unable to evaluate/treat sleep problems in children. Of note, there was variability in responses about their ability to evaluate/treat pediatric insomnia across HCP groups, with 100% (6/6) of allied health professionals, 83% (5/6) of psychologists, 80% (4/5) of nurses, and 60% (3/5) of physicians indicating that they were able to do this, but not as well as they would like.

### Research Question 1: Satisfaction

Overall, participants rated the PHS program highly in terms of satisfaction. The mean ratings for all 8 items on the SQ ranged from 3.18 to 3.45, with a response of 3 indicating “mostly satisfied” and a response of 4 indicating “very satisfied” (Table 2). The majority of participants (19/22, 86%) reported that the PHS program met their needs, they would recommend the program to colleagues, they were satisfied with the program, and they would use the program outside the study. Table 2 provides a breakdown by item.

**Table 2:** Satisfaction ratings of the PHS^a^ program by HCP^b^ type.

Satisfaction questionnaire^c^	Overall, mean (SD)	Physicians (n=5), mean (SD)	Psychologists (n=6), mean (SD)	Nurses (n=5), mean (SD)	Allied health professionals (n=6), mean (SD)
Quality of program	3.36 (0.58)	3.80 (0.45)	3.17 (0.75)	3.0 (0)	3.50 (0.55)
Content of program	3.27 (0.55)	3.40 (0.55)	3.0 (0.63)	3.0 (0)	3.67 (0.52)
Program met HCP needs	3.18 (0.59)	3.20 (0.45)	2.83 (0.41)	3.0 (0.71)	3.67 (0.52)
Recommend program to colleagues	3.41 (0.59)	3.60 (0.55)	3.0 (0.63)	3.40 (0.55)	3.67 (0.52)
Satisfaction with program content	3.27 (0.77)	3.00 (1.23)	3.0 (0.63)	3.40 (0.55)	3.67 (0.52)
Applicability of program content	3.41 (0.73)	3.60 (0.55)	3.33 (0.52)	3.40 (0.55)	3.33 (1.21)
General satisfaction with program	3.32 (0.78)	3.00 (1.23)	3.33 (0.82)	3.20 (0.45)	3.67 (0.52)
Would participants use the program outside the study	3.45 (0.67)	3.60 (0.55)	3.17 (0.75)	3.40 (0.89)	3.67 (0.52)

^a^PHS: Promoting Healthy Sleep.

^b^HCP: health care provider.

^c^All items were rated on a 4-point Likert scale, where participants were asked to indicate their level of agreement with the statements as follows: 1=quite dissatisfied, 2=indifferent/mildly satisfied, 3=mostly satisfied, and 4=very satisfied.

### Research Question 2: Overall Program Usability

Participants’ evaluations of the PHS program’s overall usability are summarized in Table 3. When asked to rate the overall program’s usability, there were generally high ratings across the 7 usability dimensions. Credibility received the highest rating, whereas findability received the lowest rating across the usability dimensions. Overall, the participants viewed the program as useful, usable, desirable, findable, accessible, valuable, and credible (ie, they gave ratings of 3 or higher on these items). When program usability ratings by type of HCP were reviewed, it was found that physicians had the highest program ratings across all 7 usability dimensions; in contrast, psychologists rated the program the lowest across all 7 usability dimensions, though they still rated the program as moderately usable.

**Table 3:** Usability ratings for the PHS^a^ program by HCP^b^ type.

Usability questionnaire^c^	Overall, mean (SD)	Physicians (n=5), mean (SD)	Psychologists (n=6), mean (SD)	Nurses (n=5), mean (SD)	Allied health professionals (n=6), mean (SD)
Usefulness	4.05 (0.84)	4.40 (0.55)	3.67 (1.0)	3.80 (0.45)	4.33 (1.03)
Usable	3.68 (0.95)	4.20 (0.45)	3.0 (0.89)	3.60 (0.89)	4.0 (1.10)
Desirable	4.00 (0.76)	4.40 (0.55)	3.83 (0.75)	3.60 (0.55)	4.17 (0.98)
Findable	3.64 (0.79)	4.0 (0.71)	3.0 (0.63)	3.60 (0.55)	4.0 (0.89)
Accessible	3.77 (0.75)	4.40 (0.55)	3.33 (0.52)	3.40 (0.55)	4.0 (0.89)
Credible	4.27 (0.70)	4.60 (0.55)	4.33 (0.52)	4.00 (0)	4.17 (1.17)
Valuable	4.00 (0.93)	4.60 (0.55)	3.50 (0.84)	3.80 (0.45)	4.17 (1.33)
Mean Rating	3.92 (0.96)	4.31 (0.56)	3.52 (0.74)	3.83 (0.36)	4.12 (1.06)

^a^PHS: Promoting Healthy Sleep.

^b^HCP: health care provider.

^c^All items were rated on a 5-point Likert scale, where participants were asked to indicate their level of agreement with the statements as follows: 1=not at all, 2=slightly, 3=moderately, 4=very, and 5=extremely.

### Research Question 3: PHS Session Usability

Generally, ratings across sessions were similar in that all means fell within the “moderate” to “extremely” usable range (Table 4). When looking at the ratings for all 4 sessions, session 4, which covered a variety of evidence-based treatments for pediatric insomnia, attained the highest ratings across all usability dimensions compared with the other sessions, except for credibility, which was high across all 4 sessions.

**Table 4:** Usability ratings for individual PHS^a^ sessions.

Usability questions^b^	Session 1 FQ^c^, mean (SD)	Session 2 FQ, mean (SD)	Session 3 FQ, mean (SD)	Session 4 FQ, mean (SD)
Usefulness	3.59 (0.80)	3.86 (0.83)	4.09 (0.75)	4.36 (0.85)
Usable	4.14 (0.71)	3.95 (0.84)	3.95 (0.79)	4.23 (0.87)
Desirable	3.95 (0.84)	4.09 (0.87)	4.09 (0.75)	4.36 (0.85)
Findable	3.55 (0.96)	3.91 (0.75)	3.95 (0.72)	4.14 (0.77)
Accessible	3.77 (0.92)	4.00 (0.62)	4.00 (0.62)	4.14 (0.77)
Credible	4.27 (0.63)	4.09 (0.81)	4.45 (0.51)	4.27 (0.83)
Valuable	3.64 (1.00)	3.82 (1.00)	4.05 (0.79)	4.14 (0.94)
Mean rating	3.99 (0.84)	3.96 (0.82)	4.08 (0.85)	4.22 (0.84)

^a^PHS: Promoting Healthy Sleep.

^b^All items were rated on a 5-point Likert scale, where participants were asked to indicate their level of agreement with the statements as follows: 1=not at all, 2=slightly, 3=moderately, 4=very, and 5=extremely.

^c^FQ: feedback questionnaire; participants completed an FQ after each program session. Ratings from each session FQ are reported in the columns above.

### Research Question 4: Program and Session Content Acceptability

Participants’ acceptability of overall program content (P-AUQ) and session-specific content (S-AUQ) is summarized in Table 5. The entire PHS program and all 4 sessions were rated highly in terms of being educational and organized, with mean ratings falling between “agree” (rating of 4) and “strongly agree” (rating of 5). Overall, the majority (12/22, >50%) of participants did not perceive the need for content to be added, deleted, or changed in the program or for any individual sessions. All participants viewed the program as educational and organized, with most participants (82%; 18/22) reporting that it was ready to be used by other HCPs. In terms of material being removed, changed, or added to the program, 70% (15/22) of participants disagreed with this.

**Table 5:** Participant ratings of acceptability of the PHS^a^ program content.

Acceptability questionnaire^b^	Program UQ^c^ M (SD)	Physicians (n=5) M (SD)	Psychologists (n=6) M (SD)	Nurses (n=5) M (SD)	Allied health professionals (n=6) M (SD)
Sessions were viewed as educational	4.59 (0.50)	5.0 (0)	4.33 (0.52)	4.40 (0.55)	4.67 (0.52)
Sessions were organized well	4.36 (0.58)	4.80 (0.45)	4.0 (0.63)	4.20 (0.45)	4.50 (0.55)
More information/material needs to be added to the program	2.90 (0.92)	2.40 (0.89)	3.0 (1.10)	2.80 (0.84)	3.33 (0.82)
Some information needs to be deleted from the program	2.55 (1.18)	2.0 (1.0)	3.17 (1.33)	2.60 (0.89)	2.33 (1.37)
Program should be changed or re-organized	2.36 (1.00)	2.0 (0.71)	2.67 (1.21)	2.60 (0.89)	2.17 (1.17)
Program is ready to be used by other HCPs	3.82 (0.73)	4.0 (1.23)	3.50 (0.84)	3.80 (0.45)	4.00 (0)

^a^PHS: Promoting Healthy Sleep.

^b^All items were rated on a 5-point Likert scale, where participants were asked to indicate their level of agreement with the statements as follows: 1=strongly disagree, 2=disagree, 3=neither agree nor disagree, 4=agree, and 5=strongly agree.

^c^UQ: usability questionnaire; mean (SD) UQ scores for all health care provider participants are reported in this column.

When ratings by HCP group were evaluated, physicians and allied health professionals had the highest ratings for the program, and psychologists had the lowest ratings for the overall program content compared with the other 3 HCP groups (Table 5).

### Research Question 5: HCP Qualitative Feedback

#### Themes

Across both the program (P-AUQ) and session-specific usability questionnaires (S-AUQ), there were 5 themes that resulted from the questions that covered the suggestions that HCPs gave to modify, add, or remove content in the program, namely: (1) modifications to increase user-friendliness, (2) increasing program interactivity, (3) program content suggestions, (4) session content suggestions, and (5) program content removal (Table 6).

**Table 6:** Program suggestion themes from the P-AUQ^a^ and S-AUQ^b^.

Qualitative themes^c^	Overall P-AUQ frequency (N=22), n (%)	Session 1 frequency (N=22), n (%)	Session 2 frequency (N=22), n (%)	Session 3 frequency (N=22), n (%)	Session 4 frequency (N=22), n (%)
Additional resources	9 (41)	7 (32)	3 (14)	4 (18)	—^d^
Program content suggestions	9 (41)	6 (27)	7 (32)	—	9 (41)
Modifications to increase user-friendliness	10 (45.5)	6 (27)	6 (27)	13 (59)	5 (23)
Program content removal	5 (23)	6 (27)	4 (18)	—	—
Increase program interactivity	—	—	6 (27)	3 (14)	—

^a^P-AUQ: Program Acceptability and Usability Questionnaire.

^b^S-AUQ: Session Acceptability and Usability Questionnaire.

^c^Themes had to be mentioned by at least 3 individual participants per questionnaire to be included. Frequency and percentage represent how many individual participants mentioned a theme in a questionnaire.

^d^Not applicable.

#### Overall Program Qualitative Feedback

Regarding qualitative feedback on the P-AUQ (Table 6), the most prevalent theme identified was the need for modifications to increase the program’s user-friendliness (10/22, 46%). This encompassed HCPs’ feedback on technical challenges, platform navigation issues, and audio-related difficulties. The next themes reported were the suggestion to include additional resources in the program (9/22, 41%) and program content suggestions (9/22, 41%). For additional resources, participants mentioned providing printable/downloadable materials, such as slides and session notes, along with compiling downloadable resource lists for HCPs and parents/caregivers. Program content suggestions included integrating content about children with neurodevelopmental disorders (NDDs) and sleep-related issues, incorporating a holistic perspective in assessing and treating sleep disorders (eg, not just looking at sleep disorders through a behavioral lens), and providing more evidence supporting the effectiveness of interventions for treating pediatric insomnia. Conversely, program content removal (5/22, 23%), such as condensing information or breaking up sessions, was the least frequently mentioned theme in the P-AUQ.

#### Session Qualitative Feedback

Regarding qualitative feedback on the S-AUQ, modifications to increase user-friendliness were referenced across all 4 S-AUQs (Table 7). Session 3 garnered the highest number of mentions of modifications to increase user-friendliness (13/22, 59%), primarily due to a minor editing error in the video session. Program content suggestions were mentioned most frequently in session 4 (9/22, 41%). Adding additional resources was the most frequently mentioned theme in session 1 (7/22, 32%). Program content removal was mentioned exclusively in sessions 1 (6/22, 27%) and 2 (4/22, 18%), with feedback focused on the scope of HCPs’ practice section in the program (eg, different HCPs’ scopes of practice when assessing and treating insomnia symptoms in children). For the theme of enhancing program interactivity, participants suggested incorporating quiz questions and more practical examples into sessions 2 (6/22, 27%) and 3 (3/22, 14%), but no mention of this theme was made in the overall program questionnaire.

**Table 7:** Illustrative quotes for HCP^a^ program suggestion themes.

Theme	Illustrative quotes^b^
Additional resources	“An option to download handouts would be really helpful for a professional to follow along as so much of this is relaying of information and no way to interact with the content.” (Psychologist 1, session 3, S-AUQ) “I would benefit from having a copy of a power point slides to accompany the speakers' oral presentations.” (Occupational therapist 1, session 1, S-AUQ) “As the program was delivered online with learning management, I would recommend to utilize the resources section of the system to upload session resources that were in the slides to that location as well.” (Recreation therapist 1, P-AUQ)
Program content suggestions	“It helps increase interest when the speaker added a few things, e.g., examples or useful information, besides the written display.” (Occupational therapist 2, session 1, S-AUQ) “Research evidence for any of the behavioural/cognitive strategies, especially if there are some that have more evidence than others.” (Psychologist 4, P-AUQ) “More on how to engage families to change behaviour.” (Nurse 2, P-AUQ)
Modifications to increase user-friendliness	“A few edits to help transition between speakers and some minor changes to the content.” (Psychologist 5, P-AUQ) “Minor editing needed, but overall session delivery and content was very good.” (Recreation therapist 1) “I also did not find the information very easily on the platform (e.g., comprehension questions were hidden).” (Psychologist 2, P-AUQ)
Program content removal	“I don't feel that the video on providers roles was beneficial in video format. A simple table would be sufficient.” (Occupational therapist 2, P-AUQ) “Too much information in some cases. First module could be broken into two smaller modules.” (Psychologist 2, P-AUQ) “Good overall. Perhaps too detailed in other sleep disorders besides pediatric insomnia.” (Psychologist 3, session 3, S-AUQ)
Increase program interactivity	“Some of the content was heavy - might be helpful to make it more interactive if possible, with polls etc.” (Psychologist 3, P-AUQ) “I don't personally retain information from listening, more of an interactive session with quiz questions throughout and applying the information may have been more helpful for me to retain the information.” (Nurse 5, P-AUQ) “I really enjoyed the cases in the sessions. More cases could also be helpful.” (Physician 1, Session 4, S-AUQ)

^a^HCP: health care provider.

^b^Illustrative quotes include the profession of the HCPs who gave the feedback, and notes whether the feedback is from the overall Program Acceptability and Usability Questionnaire (P-AUQ) or Session-specific Acceptability and Usability Questionnaire (S-AUQ).

### Research Question 6: Preliminary Program Effectiveness

A paired 2-tailed *t* test was conducted to examine changes in sleep knowledge, as measured by the SKQ scores before and after program completion. When a Shapiro-Wilk test of normality was conducted, nonsignificant *P* values were found for both variables, indicating that the data were approximately normally distributed. Participants’ preprogram SKQ scores (mean 10.27, SD 2.19) significantly differed from their postprogram scores (mean 13.50, SD 2.52; t_21_=–4.93; *P*<.001), as scores significantly increased postprogram completion. This represents a large effect size (*d*=–1.05, 95% CI –1.57 to –0.52).

A paired 2-tailed *t* test was conducted to examine changes in sleep attitudes and beliefs, as measured by SABQ scores before and after program completion. When a Shapiro-Wilk test of normality was conducted, nonsignificant *P* values were found for both variables, suggesting that the data were approximately normally distributed. Participants’ postprogram scores (mean 53.91, SD 3.88; t_21_=–2.55) were significantly higher than their preprogram SABQ scores (mean 51.18, SD 4.38; *P*=.02). This represents a moderate effect size (*d*=–0.54, 95% CI –0.99 to –0.09).

## Discussion

### Overview

The PHS program was developed to address a significant evidence-to-practice gap: children with insomnia/insomnia symptoms rarely receive evidence-based care because HCPs are not adequately trained to screen, assess, and treat these sleep problems. After completing the PHS program, participants reported high levels of satisfaction with both the program and its content. The PHS program overall and each of the 4 sessions were rated as highly usable and had high acceptability ratings from HCPs. Overall, the feedback was mostly positive, with only minor suggested changes to the program. Moreover, the PHS program demonstrated preliminary effectiveness, with improvements in HCPs’ sleep-related knowledge as well as attitudes and beliefs from before to after program completion.

When evaluating HCPs’ satisfaction with the PHS program (research question 1), over 90% of participants reported that they would recommend the program to colleagues, they would use the program outside the study, the program met their needs, and they rated the program highly in terms of quality, content, and applicability. This supports our expected results that >80% of participants would be satisfied with the PHS program. Interestingly, physicians and allied health professionals reported the highest ratings for program satisfaction overall, and psychologists reported the lowest ratings, although these were still generally positive.

Previous research suggests that parents of children with sleep difficulties often turn to primary care providers for support [35], and pediatricians frequently encounter sleep-related concerns in practice [18]. Despite this, they receive little to no comprehensive formal training in sleep assessment and treatment [37-41]. This may explain why physicians and allied HCPs in our study reported the highest satisfaction with the PHS program, given that the program provided information that was missing from their formal training and was directly relevant to their clinical work. In contrast, while many clinical psychologists receive limited training in sleep management during their education and professional development [36], they are highly trained in behavioral management, which informs the foundation of sleep intervention strategies. As such, they may not have found the program to contain novel clinical information [19]. Of note, 5 of the 6 psychologists in this study reported feeling capable of addressing pediatric sleep problems, though they still expressed a desire to improve their skills. In other health care disciplines, previous research shows that most HCPs recognize the importance of sleep health [42]; however, their awareness of its relationships to psychosocial factors remains limited [43]. These findings highlight the need for training in the screening, assessment, and treatment of pediatric sleep problems, particularly for HCPs who believe they require additional training in this area.

Participants gave high ratings across all 7 dimensions of Morville and Sullenger’s [24] UX Honeycomb framework for both the program overall (research question 2) and the individual sessions (research question 3). The mean usability rating across all 7 user dimensions was approximately 4 out of 5, with over three-quarters of the participants rating the PHS program as useful, usable, desirable, findable, accessible, valuable, and credible. Though all sessions received very similar usability ratings, session 4, which focuses on pediatric insomnia interventions (eg, behavioral strategies and medication options), received slightly higher usability ratings. This is likely due to its direct relevance to HCPs dealing with children facing sleep challenges, making this session particularly valuable, desirable, and useful. Frequency ratings from the usability and satisfaction questionnaires indicated that 2 participants were more critical in their assessment of the program: one from the psychologist group and another from the nurse group. Their responses to the questionnaires suggested that the PHS program did not align well with their content needs. To address this in future studies, it could be beneficial to administer a preprogram questionnaire, which would inquire about participants’ objectives for enrolling in the PHS program in order to assess whether the program’s content and structure are likely to meet the specific needs and expectations of each participant.

The acceptability findings (research question 4) indicated that the overall program and all 4 sessions were highly regarded for being educational and well organized, and 82% (18/22) of participants believed that the program is ready to be used by other HCPs. While the majority of participants (12/22, 55%) did not indicate the need to modify the program, some participants did provide some constructive feedback (research question 5). One key area of feedback was related to the usability of the program (eg, reducing technical difficulties and program platform issues). In e-learning programs, technical difficulties have been found to decrease motivation and negatively impact program attrition [44]. As found in previous research, usability studies identify these types of problems so that they can be addressed before larger-scale testing [24,45]. Participants also provided specific content suggestions, such as adding more information about NDDs and sleep in school-aged children. Previous research demonstrates that NDDs and insomnia in children are highly comorbid, with up to 90% of children with NDDs experiencing insomnia symptoms [37,46]. To address this feedback, we are planning to create an additional module to add to the PHS program focusing on NDDs and sleep in school-aged children. Lastly, participants suggested adding additional resources for parents and HCPs, removing some program content, and increasing program interactivity, which aligns with other research where HCPs have found programs more effective when given applicable information that they can apply directly in practice [38,39]. Incorporation of user-centered design principles through usability testing is known to enhance engagement, completion rates, and implementation in clinical practice, ultimately increasing program effectiveness [22].

Lastly, our findings revealed a significant improvement in HCPs’ scores on the SKQ and the SABQ after completing the program, indicating an increase in their sleep knowledge, attitudes, and beliefs (research question 6). This underscores that the PHS program has the potential to boost HCPs’ understanding of pediatric insomnia and how to screen, assess, and manage these sleep issues in children. The questionnaires also showed preliminary evidence that HCPs’ perspectives on sleep had changed after completing the program. This change suggests that the program also positively influenced HCPs’ perceptions of sleep modifiability, children’s responsiveness to treatment, the overall impact of sleep issues, and the nature of children’s sleep problems.

### Strengths and Limitations

A key strength of this study is that a user-centered design was used to evaluate the program by end users (ie, HCPs). Usability studies have been found to identify problems before larger-scale testing and to ensure that programs are usable to the end user, both of which are critical to user engagement with the program [24,45]. Another key strength of the study is that we recruited different types of HCPs to complete and evaluate the PHS program (ie, physicians, psychologists, nurses, and allied HCPs, such as occupational therapists, social workers, and recreation therapists), given that pediatric insomnia care is part of the clinical practice of many different types of HCPs. These HCPs often receive minimal to no training in pediatric sleep during professional training programs (eg, regular curriculum programming) or in continuing education [40-42], and as such, this program can help bridge the evidence-to-practice gap across different HCP disciplines.

There are some limitations to acknowledge in this study. Our sample predominantly identified as women, which limits our ability to generalize these findings. With this being said, approximately 70% of HCPs in Canada identify as women, so this reflects the current health care field [43]. Furthermore, we chose an a priori threshold for qualitative data analysis, meaning that comments not meeting the threshold were excluded from the final results. As a result, some important feedback may have been missed. Due to the small sample size, outlier ratings had a greater impact on questionnaire means, and we were underpowered to test whether excluding them would meaningfully change the results. Additionally, the SKQ and SABQ were author-developed and not psychometrically validated, potentially limiting their reliability and generalizability. The SKQ was based on PHS program content, which may further restrict its applicability beyond this context. Although improvements seen in sleep knowledge and attitudes after completing the program are promising, these findings need to be replicated in a larger-scale effectiveness trial. Lastly, the inclusion of HCPs who were likely interested in the content (sleep in children) and those recruited through the professional contacts of the developers of the PHS program raises the possibility of bias in their feedback.

### Future Directions

Future research should evaluate the long-term effectiveness of the PHS program by conducting assessments at 6 and 12 months after program completion to determine whether increases in sleep knowledge lead to sustained behavioral changes in clinical practice. Investigating program outcomes across various health care disciplines could determine whether effectiveness differs by professional background. Additionally, the cost-effectiveness of the PHS program could be examined in future research, as previous research on sleep education programs has found them to be economically valuable [47]. To better understand how changes in knowledge and attitudes translate into practice, incorporating interviews with HCPs to identify barriers and facilitators to integrating evidence-based sleep assessment and treatment would be valuable. Furthermore, expanding e-learning HCP training programs to address different age groups (ie, young children, youth, and adults) and specific populations (eg, NDDs) is currently in development by the research team to address these needs.

### Conclusion

In summary, this study demonstrated high satisfaction, acceptability, and usability ratings for both the overall program and the individual sessions of the PHS e-learning program from HCPs. Additionally, the study suggests that HCPs’ sleep knowledge, attitudes, and beliefs can improve following program completion. Qualitative feedback identified minor needed modifications, which will inform ongoing refinements. A health care system equipped with updated knowledge and skills regarding pediatric sleep issues is essential for providing better care to children and families facing sleep challenges. The next step involves a larger-scale effectiveness study across Canada, aiming to enhance insomnia treatment for school-aged children by better preparing HCPs. Ultimately, this user-centered online training program seeks to improve health care delivery and promote the long-term health and well-being of children in Canada [3]. From a public health perspective, an e-learning professional development program geared toward the screening, assessment, and treatment of insomnia and sleep problems in children holds immense potential to close the existing evidence-to-practice gap [45].

## Data Availability

The datasets generated and analyzed during this study are not publicly available to protect participants’ identities and information. The researchers do not have ethical approval to share the data.

## Appendix

Multimedia Appendix 1 Sleep Knowledge Questionnaire (SKQ) and Sleep Attitudes and Beliefs Questionnaire (SABS).

## Abbreviations

CSQ-8: Client Satisfaction Questionnaire-8
HCP: health care provider
NDD: neurodevelopmental disorder
P-AUQ: Program Acceptability and Usability Questionnaire
PHS: Promoting Healthy Sleep
SABQ: Sleep Attitudes and Beliefs Questionnaire
S-AUQ: Session Acceptability and Usability Questionnaire
SKQ: Sleep Knowledge Questionnaire
SQ: Satisfaction Questionnaire
