# Supplementary material for: The Promoting Healthy Sleep E-Learning Professional Development Program for Health Care Providers: Mixed Methods Usability Study
Source: JMIR Form Res. 2026 Aug 11;10:e91150. doi: 10.2196/91150 (PMC13507723; doi:10.2196/91150)
Supplement: Supplementary file 1 [file formative_v10i1e91150_app1.docx]

**Sleep Knowledge Questionnaire**

1) What is the percentage of typically-developing children that have behavioural insomnia?
a. 15%
b. 25%
c. 50%
d. 60%
2) What is the percentage of children that are diagnosed with a neurodevelopmental disorder that have behavioural insomnia?
a. 20%
b. 40%
c. 50%
d. 70%
3) What sleep stage do we stay in the longest throughout the night?
a. N1
b. N2
c. N3
d. REM Sleep
4) When does the first REM period occur?
a. 60-90 minutes after the onset of sleep
b. 60-90 minutes after the onset of sleep
c. 90-120 minutes after the onset of sleep
d. More than 120 minutes after the onset of sleep
5) The following is influenced by sleep:
a. Cognition/Learning
b. Eating Habits
c. Mental Health
d. Physical Health
e. All of the above
6) Insomnia includes the following:
a. Difficulty falling asleep, staying asleep, and/or early morning awakening
b. It is frequent and chronic (equal to or greater than 3 times per week for 3 months)
c. It is not explained by another sleep-wake disorder, medical condition, or mental health disorder
d. Non-adequate opportunity for sleeping
e. All of the above
f. A, B, and C
g. A and B only
h. A and C only
i. None of the above
7) What are some factors that can lead to insomnia?
a. Emotional health, social factors, physical health, psychiatric comorbidity
b. Environmental factors, developmental factors, medication, psychiatric comorbidity
c. Emotional health, physical health, socio-economic status, developmental factors
d. Environmental factors, social factors, medication, sleep-onset problems
e. All of the above
8) What is an actigraph?
a. A device worn on the wrist that measures your heartbeat
b. A device worn on the wrist that measures your blood oxygen levels
c. A device worn on the wrist that has an accelerometer used to measure movement
d. A device worn on the wrist that measures sleep and wake times
e. A and B only
f. C and D only
g. All of the above
9) When evaluating the sleep history of a patient, the healthcare provider should assess: sleep habits, daytime functioning, sleep symptoms, and conduct a physical examination if indicated and in their scope of practice.
a. True
b. False
10) Which is not true about sleep diaries?
a. Assesses sleep patterns across days/weeks
b. Provides a record of sleep and wake times
c. Collects information about sleep onset latency and total sleep time
d. Collects information about sleep stages such as time to get into REM sleep
11) What is the most common sleep-related breathing disorder in children?
a. Central sleep apnea
b. Obstructive sleep apnea
c. Sleep-related hypoventilation disorders
d. Sleep-related hypoxemia disorders
12) Which of the following is **NOT** an example of circadian rhythm sleep-wake disorders?

a. Delayed sleep-wake phase disorder

b. Irregular sleep-wake rhythm disorder

c. 24-hour sleep-wake phase disorder

d. Jet lag disorder

13) What percentage of children sleep walk?

a. 10%

b. 20%

c. 30%

d) 60 %

14) Which of the following are true about NREM Sleep Arousal Disorders?

a. They usually begin in the first third of the initial sleep episode

b. Their eyes are typically closed during these events

c. They are typically long, lasting 20-40 minutes long

d. Complex behaviours can occur from sleep, including confusional arousal, sleep terrors, and sleep walking

e. All of the above

f. A and B are correct

g. C and D are correct

h. A and D are correct

15) What are some red flags to look for during assessment of sleep problems in children?
a. Any sign or symptom of Obstructive Sleep Apnea
b. Symptoms of a mental health disorder
c. History from parents of restlessness or sleep related movements
d. Parasomnias
e. All of the above
f. A and B only
g. A, B and C only
h. B and D only
16) Which of these is **NOT** one of the four best practice interventions for pediatric insomnia?
a. Medication
b. Neurofeedback
c. Specific Behavioural Interventions
d. Healthy Sleep Practices
e. Psychoeducation
17) What is the percentage of children that have sleep problems that are prescribed medication when parents bring their concern to the child’s physician?

a. 50%

b. 60%

c. 80%

d. 95%

18) Which of the following is the correct definition for Graduated Extinction?

a. Involves parent putting child into their sleeping space awake, leaving the room, and going back to provide comforting at regular or scheduled intervals

b. Leaving child in bed or bedroom until they fall asleep without any parental presence or checks

c. Involves the parent putting child into their sleeping space awake and staying in the room until the child falls asleep but gradually decreasing the amount of interaction over time

d. Put child to bed ~30 minutes later than when child usually falls asleep – the idea is that they will be more tired than usual and fall asleep quicker

19) Cognitive Strategies are used to address non-productive beliefs about sleep, such as misinformation about sleep and faulty beliefs.
a. True
b. False
20) Which of the following are **FALSE** regarding prescribing medication for sleep problems in children?
a. There are no approved medications for pediatric insomnia.
b. The use for pharmacotherapy in insomnia is based on excellent evidence from randomized controlled studies of pediatric insomnia.
c. Physicians may prescribe medication not only for the child, but also so parents or other family members can sleep
d. Timing when prescribing is important – avoid giving medication during enhanced alertness in evening
21) There is evidence in the literature of the effects of long-term melatonin use in pediatric insomnia patients.
a. True
b. False

**Sleep Attitudes and Beliefs Scale (SABS)**

The following questions ask about your beliefs about sleep and behavioural sleep problems in children. Please indicate the extent to which you agree with each item.

| 0 (Do not agree at all) | 1 | 2 | 3 | 4 (Very much agree) | Prefer not to answer |
| --- | --- | --- | --- | --- | --- |

*Row headings (items):*

1. Sleep problems in children are the result of poor sleep routines

2. Solving children’s sleep problems mostly takes common sense

3. Most children’s sleep can be modified so that there are no sleep problems

4. Healthcare providers are able to change children’s sleep habits

5. Childhood sleep problems can be solved through changing the child’s sleep habits

6. Healthcare providers can’t change their children’s sleep patterns (reverse scored)

7. Children’s sleep patterns cannot be modified

8. Treatments for sleep problems are not effective in children

9. No matter what a parent does, a child will still have sleep problems

10. Healthcare providers don’t have a lot of success in changing their child’s sleep patterns

11. Sleep problems affect other areas of children’s physical and mental development

12. Sleep problems in children can cause daytime behaviour problems

13. Children with sleep problems do less well at school

14. A child’s sleep problems can have a big impact on the whole family

15. Children’s sleep problems are due to underlying medical conditions

16. Sleep problems are a result of neurological causes

17. Childhood sleep problems are biological in nature
